# Experiences of participating in a preoperative comprehensive geriatric assessment and care intervention among frail older adults before colorectal cancer resection surgery

**DOI:** 10.1186/s12877-025-05922-9

**Published:** 2025-05-05

**Authors:** Kristina Åhlund, Lena G. Larsson, Niklas Ekerstad, Maria Normann, Mattias Prytz, Anette Johnsson

**Affiliations:** 1 Department of Research and Development, NU Hospital Group, Trollhättan, Sweden; 2 Department of Health Sciences, University West, Trollhättan, Sweden; 3 Department of Orthopaedics, Institute of Clinical Science, Sahlgrenska Academy, University of Gothenburg, Gothenburg, Sweden; 4 Research, Education, Development and Innovation, Primary Health Care, Region Västra Götaland, Gothenburg, Sweden; 5 Department of Health, Medicine, and Caring Sciences, Unit of Health Care Analysis, Linköping University, Linköping, Sweden; 6 Department of Surgery, Institute of Clinical Sciences, Sahlgrenska Academy, University of Gothenburg, Gothenburg, Sweden; 7 Department of Surgery, NU-Hospital Group, Region Västra Götaland, Trollhättan, Sweden

**Keywords:** Colorectal cancer, Comprehensive geriatric assessment and care, Frailty, Interview study, Older adults, Preoperative

## Abstract

**Background:**

Comprehensive geriatric assessment (CGA) and care has shown benefits for frail older adults across various care settings. However, its integration into routine care within a surgical context remains limited. An ongoing randomised controlled multicentre study will evaluate the effects of a period of preoperative optimisation (up to eight weeks), involving interprofessional CGA and care, in addition to routine care before colorectal cancer resection surgery. If this approach proves favourable, it could potentially be incorporated into routine surgical care. To facilitate implementation, it is crucial to explore and understand participants’ perceptions of taking part in a preoperative CGA and care intervention.

**Aim:**

To describe how frail older adults with colorectal cancer experience participation in a preoperative CGA and care intervention.

**Methods:**

This qualitative, descriptive study was part of a randomised controlled multi-centre study. In total, 20 semi-structured interviews were conducted with frail older adults undergoing a CGA and care intervention before colorectal cancer surgery. The data were analysed using inductive qualitative content analysis.

**Results:**

Frail older adults with colorectal cancer experienced participation in preoperative CGA and care as an integral part of an intervention. They adopted an opportunity mindset when deciding to participate. Throughout the intervention, they observed the team working collaboratively and actively involving them in the optimisation process, enhancing their readiness for surgery by the end of the period.

**Conclusions:**

The findings indicated that frail older adults with colorectal cancer viewed the preoperative CGA and care intervention as a meaningful opportunity for improvement and a chance to extend life. Their active involvement and the collaborative efforts of the care team during the intervention were crucial in enhancing their understanding, manageability, and readiness for surgery. They valued the opportunity to make active choices when appropriate and appreciated having the right to delegate decisions to healthcare professionals. From a frail older adult’s perspective, team-based approaches in preoperative care, such as CGA and care, offer benefits in terms of involvement and satisfaction. However, the thorough evaluation of postoperative outcomes remains necessary.

**Trial registration:**

OSF registry: https://osf.io/ch49n, registered on Sep 04, 2023.

**Supplementary Information:**

The online version contains supplementary material available at 10.1186/s12877-025-05922-9.

## Background

Today, more people are reaching old age than in previous generations and forecasts indicate an ongoing increase [1]. Old age combined with morbidity and disability is associated with frailty, a condition commonly used as an indicator of biological age [2, 3]. Frailty involves diminished physiological reserves and decreased resilience, increasing the risk of deterioration in case of acute stress, such as illness and surgery [4]. Commonly used frailty definitions are based on either Fried’s phenotype model [5], which emphasises physical aspects, weight loss, and fatigue, or Rockwood’s deficit of accumulation model [2], which considers the various diseases and disabilities a person has accumulated throughout life. Frailty typically affects a person’s ability to perform activities of daily living and can result in increased dependence, diminished autonomy, elevated fall risk, hospitalisation, institutionalisation, and mortality [4, 6]. Previous research has demonstrated a strong association between frailty and adverse outcomes in acute clinical settings [7].

Colorectal cancer (CRC) ranks as the third most common malignancy globally [8]. It predominantly affects the elderly population, and in Sweden the median ages at diagnosis are 72 years (rectal cancer) and 75 years (colon cancer) [9]. In most cases, surgery is the preferred initial treatment, sometimes in conjunction with radiotherapy and/or chemotherapy. In the absence of planned surgical treatment, frail older adults face a significant risk of requiring emergency surgery. Consequently, most patients, regardless of age, are eligible for elective procedures [10]. Advanced age alone is often considered a blunt measure of risk assessment in relation to surgery, so factors associated with biological ageing, such as frailty, comorbidities, and physical deterioration, are instead regarded as more decisive [11].

In relation to CRC surgery, old age and frailty have been associated with severe postoperative complications, prolonged hospital stays, higher readmission rates, and increased short- and long-term mortality [12–14]. The picture may seem bleak, but it is important to remember that frailty is a dynamic condition where deterioration is common, but the individual course may vary [15]. If the individual’s inherent resilience is enhanced prior to surgery, it can potentially influence postoperative outcomes. Thus, with adapted treatments, such as appropriate medication, nutrition and exercise therapy, the prognosis may be improved [16–18]. Therefore, it is advantageous to be aware of the frailty status of older adults, as this knowledge can provide opportunities to optimise their condition, mitigate the progression of frailty, and, ideally, enhance the patient’s resilience to external stressors [18, 19].

For frail older adults, interprofessional and holistic preoperative programs are considered especially beneficial [20, 21]. In this group of people, challenges arise due to their reduced capacity to adhere to treatment plans. Research has shown that patients with two or more concurrent illnesses are at a significantly higher risk of difficulties in comprehending health information than are those without multimorbidity [22]. Adherence to instructions depends on the person’s understanding of the programme and ability to perform the recommended activities. To overcome these challenges, research emphasises the importance of personalised information and support [21]. Health consultations with frail older adults often need to be longer and conducted in a calm setting, allowing sufficient time for patients to reflect on critical medical decisions. Frail older adults may also require additional motivational support to participate in shared decision-making, compared with younger and healthier individuals [23].

Comprehensive geriatric assessment (CGA) and care is considered best practice for managing frail older adults with complex conditions [24, 25]. It has been shown to significantly increase the chance of remaining alive and in one’s own home at follow-up after in-hospital care [26], and it has shown promising results in reducing medical complications in the perioperative context [27]. CGA and care is a person-centred care model that captures multiple perspectives through interprofessional collaboration, including geriatrics, medicine, nursing, physiotherapy, and nutrition. Each healthcare professional uses standardised assessments to evaluate the patient’s needs and abilities, and then the team collaboratively develops a coordinated plan to achieve shared goals.

An ongoing randomised controlled multicentre study (described below as the Colorectal Cancer Frailty Study) is evaluating the effects of a period (up to eight weeks) of preoperative optimisation through CGA and care, in addition to the standard enhanced recovery after surgery (ERAS) programme, in frail older adults with CRC cancer [28]. If the method demonstrates improved outcomes, there is potential to implement this approach in routine surgical care. To better understand how preoperative CGA and care is perceived by participants, the patient perspective also needs to be evaluated. This study therefore aims to describe how frail older adults diagnosed with colorectal cancer experience participation in a preoperative CGA and care intervention.

## Methods

Data reporting in this study follows the consolidated criteria for reporting qualitative research (COREQ) [29].

### Design

This study employs an exploratory and descriptive design [30], selected for its appropriateness for gaining an in-depth understanding of experiences important to participants with frailty and colorectal cancer participating in a preoperative CGA and care intervention. The data consist of semi-structured interviews analysed using inductive qualitative content analysis, focusing on both manifest and latent content [31].

### Setting

This study is part of the research project “Effect of comprehensive geriatric assessment for frail elderly adults operated for colorectal cancer – the colorectal cancer frailty study” [28], which is being conducted at three healthcare units in western Sweden, i.e., one university hospital and two county hospitals, in total serving a catchment area of approximately 1.2 million inhabitants. The main study was approved by the Swedish Ethical Review Authority (Dnr: 2019–05340), with additional approval obtained for this sub-study (Dnr: 2021–06220 - 02). The main study was preregistered at ClinicalTrials.gov (NCT04358328) and this study at the OSF registry (https://osf.io/ch49n).

### Preoperative CGA and care intervention

In the Colorectal Cancer Frailty Study [28], patients who consented to participate in the study were randomised to the control group (receiving standard care) or the intervention group. The frail older adults in the intervention group received preoperative CGA and care in addition to standard care, beginning with a scheduled “intervention day”. On this day, patients, and any accompanying relatives, met individually with an internal medicine physician with geriatric medicine expertise, a study nurse, a physiotherapist, and a dietician. Additionally, an occupational therapist was involved at one study site and a pharmacist at another. The interprofessional team conducting the assessments was trained in the CGA and care concept before the study’s initiation and could, if needed, consult local experts throughout the study.

A representative of each profession clinically assessed the frail older adult using standardised screening tools, and the team then developed a joint individualised care plan, tailored to the person’s needs. The team members had regular contact with the patient and the team met weekly to revise the plan. During these meetings, the potential benefits of the intervention were weighed against the risk of delaying surgery. To optimise preoperative preparation, the intervention was adapted based on each person’s assessed needs, adherence and capability and could, for example, include additional medical examinations, laboratory tests, telephone follow-ups, exercise programs (home-based programs or supervised in a gym), dietary recommendations, and the prescription of various nutritional supplements. Although the typical time from diagnosis to surgery was two to six weeks, it was agreed to extend the time to eight weeks if needed, to maximise the intervention’s effectiveness. However, the goal remained to prepare the frail older adults for surgery as quickly as possible without compromising medical safety. The postoperative phase was managed similarly in both groups [28].

### Participants

The inclusion criteria for the Colorectal Cancer Frailty study required participants to be aged 65 years or older, diagnosed with potentially curable colorectal cancer, and assessed as frail (CFS score 4–8) according to the Clinical Frailty Scale- 9 (v2.0) [2, 32]. Patients in a palliative situation, requiring urgent/emergent surgery, experiencing terminal illnesses with an estimated life expectancy of under six months (CFS score 9), unwilling to participate, or unable to understand the study information were excluded. Additionally, in this study, patients with cognitive or communicative impairments that made participation in an interview difficult were excluded.

To capture a diverse range of perspectives and to enhance the credibility of findings, a strategic sampling procedure was employed to include both men and women from at least two study sites, representing a range of ages, degrees of frailty, living situations, and stages of both colon and rectal cancer. The target was to recruit 15–20 participants, as this was deemed sufficient to achieve the goal of capturing both common patterns and unique variations relevant to this context [31]. Frail older adults who met the inclusion criteria were informed about the study, verbally and in writing, with information provided to the patients in the informed consent form, by a research nurse at the surgical clinic, consecutively as they were enrolled in the intervention group in the Colorectal Cancer Frailty study. After giving their written consent, the participants were contacted by the first author (KÅ) to arrange an appointment for the interview.

### Data collection

The data collection took place from April 2022 to December 2024. Twenty-three frail older adults were invited to participate in the study, of whom 20 gave their informed consent to participate and completed an interview (ten women; median age 83 years, range 70–93). Please see Table 1 for the characteristics of the study population.

**Table 1:** Characteristics of the study population

*N* = 20	
Age, years, median (min–max)	83 (70–93)
Female, *n* (%)	10 (50)
Type of cancer	
− Colon, *n* (%)	15 (75)
− Rectum, *n* (%)	4 (20)
− Colon + rectum, *n* (%)	1 (5)
Tumour stage	
− I, *n* (%)	5 (25)
− IIA and IIB, *n* (%)	7 (35)
− IIIB and IIIC, *n* (%)	8 (40)
CFS, median (min–max)	4 (4–6)
− CFS 4, *n* (%)	13 (65)
− CFS 5, *n* (%)	5 (25)
− CFS 6, *n* (%)	2 (10)
Living situation	
− Rural, *n* (%)	4 (20)
− Village, *n* (%)	4 (20)
− Town, *n* (%)	9 (45)
− City, *n* (%)	3 (15)
− Nursing home/Assisted living facility, n (%)	1 (5)
− Living alone, *n* (%)	9 (45)
− Home care recipient, *n* (%)	3 (15)
Waiting time, median (min–max)	41.5 (28–113)
Interview approach	
− Face-to-face, *n* (%)	13 (65)
− Telephone, *n* (%)	7 (35)
Length of interview, minutes median (min–max)	39.5 (24–71)

*CFS* Clinical Frailty Scale

Tumour stage = UICC stage grouping, based on cTNM classification

Waiting time = days from inclusion until the day before surgery

Participants were allowed to choose the time and place of the interviews, either face-to-face in their homes or at a suitable location in the hospital. Alternatively, interviews could be conducted by telephone, at the participants’ request. In cases in which relatives wished to be present, they were informed that the primary focus was on the participant’s experience. A semi-structured interview guide was developed, for this study, to obtain the participants’ experiences of participation in a preoperative CGA and care intervention. For an English version of the interview guide, please see the (supplementary file). During the interviews, the guide was used as a reminder, emphasising areas to discuss, and was associated with follow-up questions used to elaborate the answers. To further ensure that the focus of the interviews was on the participants’ experiences of participating in the preoperative CGA and care intervention, the interviewer repeated the aim of the study before each interview and shortly reminded that the focus was on the participant's narrative about their experiences of participating in the intervention, that all data would be presented in anonymised form and that the information provided would not impact future care and treatment. The interviews lasted 24–71 (median 39.5) minutes and were audio-recorded. Towards the end of data collection, the information that emerged was similar and repetitive.

To enhance reflexivity, researchers must be mindful of their preconceptions about the phenomenon studied when interviewing and analysing [30]. The author’s team included experienced clinicians and researchers from different professions: two surgeons, one cardiologist and internal medicine physician, one registered physiotherapist, and two registered nurses. The authors, i.e., KÅ, MP, MN, and NE, enrolled patients in the main study and KÅ, AJ, and LGL conducted the interviews. KÅ was also involved in the main study and sometimes performed assessments according to that study’s protocol. No interview was conducted by any author involved in the care or rehabilitation of the participant in question.

## Data analysis

Inductive qualitative content analysis [31] was used to analyse the data, focusing on both manifest and latent content. Manifest content concerns the obvious content, what is explicit in the text, while latent content entails interpretation to identify the underlying message of the text [33]. The interviews were first transcribed verbatim, then the analysis involved reading and rereading the interviews before meaning units, based on study aim, were identified and documented in computer tables, condensed into descriptions, and labelled with codes (Table 2). The codes were grouped by similarity, and then organised into sub-categories and then categories, resulting in themes that captured the overall meaning. An iterative process of moving between data and categories occurred, with all authors discussing and refining the analysis until consensus was reached. Quotations were included in the results to confirm the interpretation of the participants’ descriptions [31].

**Table 2:** Example of the analytical process

**Condensed units**	**Code**	**Subcategory**	**Category**
If I don't undergo this surgery, I will likely die in 6–7 months. Through the project and surgery, I have a chance for life afterwards	Surgery is a chance to live longer	A chance to extend life	Adopt an opportunity mindset
I would have felt different if I had not received all this. I feel a bit privileged	A sense of privilege due to the opportunity they received	Feeling privileged	

During the process of analysis, KÅ, AJ, and LGL independently performed the coding and a preliminary analysis, the results of which were compared and, when necessary, discussed, until consensus was reached. After this, the results of the preliminary analysis were presented to and discussed with NE, MP, and MN.

## Results

**Fig. 1:**
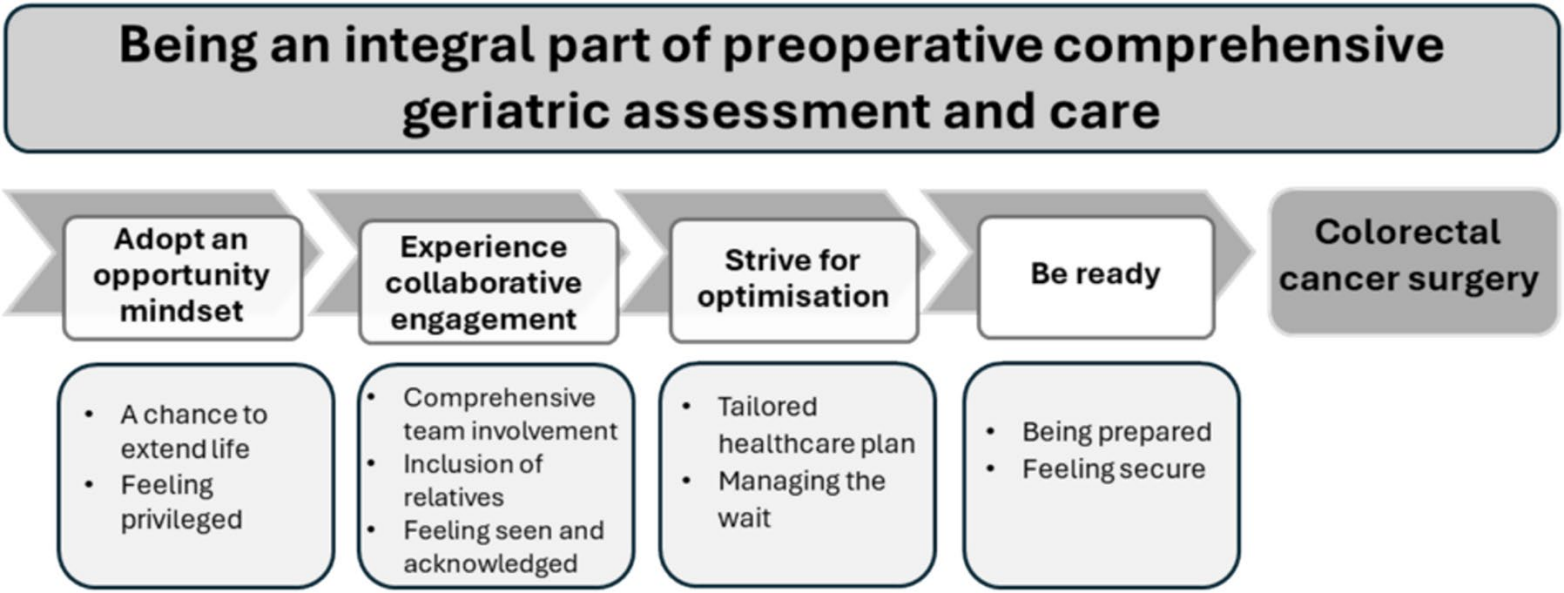
A summary of overall theme, categories, and sub-categories

The findings are presented in terms of an overall theme with four main categories and nine sub-categories (Fig. 1).

### Being an integral part of preoperative CGA and care

The analysis revealed that frail older adults diagnosed with colorectal cancer adopted an opportunity mindset when participating in a preoperative CGA and care intervention. During the intervention period, they experienced the team working collaboratively and actively involving them in striving for optimisation, which contributed to their readiness for surgery by the end of the period (Fig. 1).

### Adopt an opportunity mindset

Adopting an opportunity mindset covers participants experiencing a chance to extend their lives and at the same time feeling privileged.

#### A chance to extend life

Receiving a period of preoperative CGA and care and the opportunity to undergo surgery was portrayed as offering the possibility of prolonged life. It brought a sense of hope, despite the cancer diagnosis, frailty, and advanced age. In some cases, participants described difficulties in understanding the purpose of the intervention, while describing the positive experience of being able to try something new. It also emerged that participants recognised the benefit of their involvement beyond themselves, such as being able to help others in similar situations in the future:

If I don’t go through with this operation, well, then I’ll die in about six to seven months, or maybe in a year, in great pain. But if I do have the operation, I’ll have a chance afterwards. Then I might live for another five or six years. Though I’m not that old, of course – I’m only 93, nearly 94 [winks]. … It’s really about seeing how such a frail elderly person copes – with eating, training, and all that … I mean, how to manage others who come after, who are in the same situation before an operation. You can’t just assume they’ll cope – you need to test it on a guinea pig, ha ha. (ID 3)

The participants expressed a desire to manage the initial period after the operation as independently as possible. This also involved a desire to continue living their lives, carrying out their daily tasks and other activities they had previously been able to do:

I want to resume the life I had before. Yes, I hope that will be the case. I mean, so that I don’t end up with any lasting damage. (ID 10)

#### Feeling privileged

The participants expressed feeling privileged at having been part of the intervention, which included proactive counselling, additional examinations, and personalised treatments with professionals from various fields:

I have met the staff, and I have developed trust in them. Yes, perhaps I would have felt differently if I had not received this. So, I feel a bit privileged. (ID 1)

Furthermore, participants described experiencing trust in their interactions with the professionals they met. One example was being given a direct phone number to the relevant person, which made it easier to get in touch when needed. Simply knowing that they would not have to call the switchboard, with the risk of being connected to the wrong person, reduced the need to call and reinforced the sense of privilege:

The usual phone number doesn’t get you through, but being able to call, ask questions, and get through – and above all, get an answer, without being referred to someone else [is appreciated]. It’s more about knowing that I can get in touch if needed, that I can reach someone. That’s enough – I might not use it or need it, but just having the option is reassuring. (ID 17)

### Experience collaborative engagement

Collaborative engagement involves mutual engagement between the team, the frail older adult, and their relatives, to achieve a common goal. The participants felt seen and acknowledged, felt that their relatives were involved, and that all team members were well informed.

#### Comprehensive team involvement

The participants observed that staff from various professions worked together as a team. They noted that everyone was well informed about one another’s work and clearly understood their roles and responsibilities within the team. For instance, team members had regular follow-up meetings with the participants for various reasons. The coordination within the team was led by a designated nurse, who had more frequent contact with the participants. Additionally, the participants noticed that team members kept one another informed of developments such as test results, nutritional issues, and mobility limitations. Many participants felt that the professionals they encountered were committed and they appreciated the extra level of care that resulted from the team’s collaborative efforts:

The physiotherapist called me one day when I was sick. He told the others. They called in any case, but by then they already knew I had a cold. I found that they communicated very well with one another, and they also had weekly meetings in which they talked about me. (ID 10)

#### Feeling seen and acknowledged

All participants had previous healthcare experience, but now they felt recognised in a new and different way. The team fostered a sense of involvement, making them feel like part of a whole working towards a shared goal. Participants trusted that everyone knew what they were doing, that they would be pleasant and do their best:

Right from the start, I was treated well. It wasn’t just about being old and frail – I was addressed in the way that I wanted to be addressed. She [i.e., the nurse] kept me informed so well. In some ways, it felt very caring. I think it’s fantastic that she took the time to do that. (ID 4)

#### Inclusion of relatives

When a relative was present, their involvement was described as providing support during discussions of treatment, but the participants also described their relatives as involved in the planning process. This could include assistance with transportation and various types of bookings, such as scheduling appointments at health centres for blood tests and coordinating medical visits:

What I’ve missed is getting some help with scheduling blood test appointments, because it’s not easy to sit there and scroll through and manage 1177 [i.e., the digital booking system]. We’ve managed both times, but I’ve had to book the tests myself. It’s gone well, but I think it can be difficult for many people to get blood test appointments that align with other visits. (wife to ID 16)

The participants said that relatives who were present during various appointments assisted by taking notes on the information provided by the healthcare professionals. This helped them remember important details related to the preoperative intervention:

I had two of my daughters with me, so they helped me listen, which was reassuring. They listened to what was being said, and they could help me by reminding me, saying, “This is how it was”. (ID 14)

Relatives were also included to ensure that the frail older adult’s needs and wishes were considered. Although this provided reassurance, it could also be experienced as a burden and a source of anxiety, particularly when family members were also elderly or in poor health:

No, it’s just so that they don’t send me home too quickly but instead take care of me and do the necessary tests. And that they don’t burden her [i.e., my wife] with a lot of work – she’s sick herself. (ID 11)

### Striving for optimisation

The intervention was regarded as striving to optimise health through care initiatives that enabled participation in a tailored, individualised healthcare plan. The participants felt that they were performing to the best of their abilities; however, they also perceived the intervention as intense, due to transport issues and the numerous tasks required during the preoperative period.

#### Tailored healthcare plan

The participants described having to undergo various examinations and tests. Discussions with professionals from various fields facilitated the inclusion of participants’ preferences in the process, forming the foundation for creating a tailored, individualised healthcare plan. This plan included assessments, treatment, and support based on the participants’ medical status, perceived health, physical ability, and life situation. For example, they could easily be offered additional examinations and adjustments of the medication regime:

He [i.e., the physician] listened [to my heart] and arranged for me to go in for an ultrasound. (ID 14)

Unfortunately, I take many medications, and they have been changed several times. I don’t know why, but they have been monitoring everything very closely. (ID 11)

The opportunity to make active choices when possible was appreciated by the participants, such as selecting from available nutritional drinks and adapting exercise programmes according to their own motivation and ability:

As for the drink, I thought it was good, and everyone allowed me to be involved in the planning. It has been a great help to me. (ID 7)

Then I was supposed to go for walks, but I can’t, because I can’t get outside. So instead, I walk back and forth here inside. And I do exercise every morning with Sofia [on the TV]. (ID 1)

The participants described taking their responsibilities seriously. They understood the importance of doing what they could to maintain physical function and build strength, even though it was sometimes challenging:

He [i.e., the physiotherapist] has made it so that I exercise every day. I’m to use a walker and a chair for my exercises. And then I go for a walk every day. Previously, I did my walks. Now, I have added these exercises one to three times a day. Well, you do the best you can. (ID 15)

There were also instances when participants chose not to be involved in planning their care, which meant a conscious delegation of responsibility to the team. They trusted the team’s expertise and delegated decision-making to them:

I haven’t felt the need to influence anything. I’ve found the tips and advice to be really good. I’ve tried to follow the advice. I haven’t taken any initiative myself – I’ve just followed what I’ve been given. I’m satisfied, I think it’s great. (ID17)

#### Managing the wait

Having to keep track of many dates and appointments could feel overwhelming. However, by having a clear overview of what was happening and when, the time seemed to pass more quickly and the waiting period before the surgery became more manageable. Staying active and having something to do also created a sense of structure in everyday life and helped the participants maintain energy levels. It became a way to stay engaged and focused, which in turn reduced the feeling of simply waiting for the operation:

There have been many dates to keep track of and many contacts, so I’ve made a list to remember everything. I’ve been doing this pretty much since the beginning of May. But I think it’s been very good, because it somehow shortens the waiting time. It helps to keep up with all the contacts, and it feels like time goes by a bit faster when you have something to do. (ID 17)

### Be ready

“Be ready” involved the participants feeling prepared, strong, well informed, and secure before the operation. During the preparation period, a sense of safety was established, which made it easier for the participants to feel comfortable about the surgery.

#### Being prepared

The frail older adults described the preoperative preparations as comprehensive, encompassing both physical and mental aspects. They believed that the structured, step-by-step process, which included continuous updates and thorough information, fostered a sense of coherence and understanding, which made the participants more prepared:

I can see that there’s a common thread running through everything they do. I get that. I think the healthcare system seems much better now, much more thorough than it used to be. I’m just very grateful for all the good information I’ve received. I never thought healthcare could be this good. (ID 15)

Participants explained that exercise programmes and daily physical activity, such as walking, combined with a healthy diet, supplemented by nutritional drinks and medications, gave them the necessary energy and helped build strength:

I have a stomach ache, but something told me that I am a bit better now. And what was better? Well, it was my strength, simply. I had received some blood and had become stronger, compared with before, when I could barely live. (ID 4)

The preoperative period also provided time for mental preparation and reflection on the risk that life could end. Participants said that it was important to take care of practical matters, such as removing the boat from the lake and organising paperwork at home:

Being part of the study has been really good for me mentally. It’s helped me feel more prepared. (ID 7)

It’s tough. I need to go through all the albums and everything to see what should be thrown away. Some things might be interesting for future generations. And then, there’s my family tree research, which I’ve spent hundreds of hours on. There’s a lot of that too. I want to try to sort it out and write things down. That’s how it is. (ID 12)

#### Feeling secure

The preoperative intervention helped participants feel safe and well cared for throughout the intervention. Encounters with professionals from various fields reassured them, creating a sense of acknowledgement and of being in safe hands:

It felt safe. I was in good hands somehow. (ID 15)

I thought it was good. They had everything under control, and I got to understand all the aspects. It affects me physically, like the food I’m a bit clueless about, and then how I’m feeling. Yeah, the intervention covers everything. The whole package, you know. (ID 10)

Participants also expressed a sense of existential security rooted in their personal beliefs. This provided an inner sense of assurance that things would go well, regardless of what happened in connection with the surgery and cancer treatment:

If I may be a little serious, then it will go as it goes. I live my life, and I have done so since I was a young boy, believing that there is joy beyond death and a future filled with song. It helps me. It gives me strength. (ID 16)

## Discussion

This study provides a deeper understanding of how frail older adults experienced a period of preoperative CGA and care before colorectal cancer surgery. Overall, the findings capture the participants’ perception of being an integral part of a CGA and care intervention, including aspects related to adopting an opportunity mindset, experiences of collaborative engagement, and striving for optimisation so as, ultimately, to be ready for surgery.

Previous research has found that older patients may express reluctance to be identified as frail, as the term is often associated with a negative image of aging, including functional decline and dependency, with adverse consequences [34]. However, the findings of this study showed that the participants appreciated the chance to undergo an assessment that evaluated their potential for improvement when identified as frail.

Participants appreciated the healthcare staff’s kindness and dedication and felt that the CGA and care team’s collaboration led to higher-quality care. They believed that the team’s collective effort enhanced their experience, making them feel seen and valued, which is also supported by research [35]. Participants experienced the team as working together and being continuously up to date with current events regarding them, which was reassuring and allowed participants to trust the staff and their recommendations. These observations underscore the value of interprofessional collaboration in delivering high-quality patient care, as it appears to positively influence patient satisfaction. In addition, recent research [27] shows that patients undergoing perioperative CGA and care in association with colorectal cancer surgery experience a notable reduction in postoperative complications.

To facilitate good adherence, preoperative programmes for frail older patients should be tailored to their personal needs [36]. In this study, participants valued the ability to make active choices, such as selecting appropriate exercises and preferred foods, which helped them adhere to the recommended treatment. However, there were occasions when the participants felt reassured by expert assistance. The participants particularly appreciated receiving good information and being invited to discuss various options, although the ultimate decision-making was left to healthcare professionals. The context was important for the ability to receive information and actively choose to delegate decisions [23]. The CGA and care intervention appeared to contribute to a sense of involvement, even when decisions were delegated to others.

The involvement of relatives in healthcare visits was emphasised by the participants. Relatives helped with practical tasks such as coordinating medical visits and transportation, which eased the logistical burden on participants. When needed, they also provided psychological support. These findings align with those of previous research [37], highlighting the crucial role of relatives in the care of frail older adults by managing care expectations, understanding the patient’s condition and care plan, and providing support during periods of functional decline.

Transportation to the healthcare centre or hospital for the additional visits required by the CGA and care intervention was sometimes perceived as cumbersome, especially for participants living in urban areas with heavy traffic and needing to change public transportation mode from tram to bus to get to the appointment. To implement the concept on a larger scale in different environments, it would be crucial to assess the significance of examinations and physical visits and, if possible, coordinate them optimally to facilitate participation based on the individual patient’s needs.

The participants reported being well informed about their illness and stated that they had received clear preparation for the upcoming surgery. These findings can be linked to the participants’ sense of coherence (SOC), which reflects a person’s view of life and ability to respond to stressful situations through comprehensibility, manageability, and meaningfulness [38]. SOC refers to the participants’ ability to identify both their internal and external resources and to utilise them in ways that promote health and well-being. By understanding what to expect and feeling involved in the process, the participants experienced both comprehensibility and manageability, which reduced stress and provided a more secure experience during their care. In cancer care, guidelines and the general perception stipulate that the waiting time for surgery should be minimised. Research has shown that waiting passively may lead to increased anxiety, fear, and feelings of isolation [39]. However, in connection with the present preoperative optimisation intervention, participants did not express any anxiety related to the waiting time. They said that they used their time productively and that it felt as though the time passed more quickly when they were occupied with meaningful tasks, which also can be related to SOC [38].

Another important aspect described by participants was existential preparation for the possibility that life might end as a consequence of either the surgery or the cancer itself. Participants experienced their faith as strengthening their belief that everything would be all right, regardless of the outcome, contributing to a sense of existential security. A systematic review [40] shows that lack of spirituality is linked to demoralisation and highlights the healthcare system’s role in addressing the need for spiritual support. Although the CGA and care intervention did not primarily include a spiritual dimension, the supportive conversations with the nurse contributed to a sense of security and of being in good hands.

To ensure trustworthiness, qualitative studies must be carefully designed [33]. To capture a diverse range of perceptions regarding participation in a preoperative CGA and care intervention, a strategic sampling procedure was used, including both men and women of various ages, and living situations, representing two study sites. To ensure authenticity, patients were informed that the interviewer would not be involved in their care. The diverse professional backgrounds of the author group were a strength, as multiple perspectives relevant to CGA and care could be considered. The group has been aware that researchers with specific knowledge of frailty and CGA and care should be mindful of their preconceptions during interviews to ensure that the participants'own narratives of their involvement in the intervention are captured. Furthermore, to avoid over-interpretation, the analysis process was characterised by a coding process that closely followed the text, with interpretation occurring only in the final step to formulate themes. For credibility, the “Results” section includes quotations that reflect the essence of the themes, and the analysis was conducted through an iterative triangulation process until all authors reached consensus.

## Conclusions

The findings indicated that frail older adults with colorectal cancer viewed the preoperative CGA and care intervention as a meaningful opportunity for improvement and a chance to extend life. Their active involvement and the collaborative efforts of the care team during the intervention were crucial in enhancing their understanding, manageability, and readiness for surgery. They valued the opportunity to make active choices when appropriate and appreciated having the right to delegate decisions to healthcare professionals. From a patient perspective, team-based approaches in preoperative care, such as CGA and care, offer benefits in terms of involvement and satisfaction. However, thorough evaluation of postoperative outcomes remains necessary.

## Supplementary Information

Supplementary Material 1: Interview guide.

Supplementary Material 2: Consolidated criteria for reporting qualitative studies (COREQ): 32-item checklist.

## Data Availability

Data is provided within the manuscript or supplementary information files.
