# Supplementary material for: Experiences of participating in a preoperative comprehensive geriatric assessment and care intervention among frail older adults before colorectal cancer resection surgery
Source: BMC Geriatr. 2025 May 5;25:310. doi: 10.1186/s12877-025-05922-9 (PMC12051325; doi:10.1186/s12877-025-05922-9)
Supplement: Supplementary file 1 — Supplementary Material 1: Interview guide. [file 12877_2025_5922_MOESM1_ESM.docx]

**Interview guide**

1. **Opening question**

- Would you like to tell me how all of this started? How did you notice that you were getting sick and what happened next? … Please explain a bit more. It's valuable that you share this.

**2.** **Key questions (experience with CGA intervention)**

- Describe how it was the day you met the nurse, physician, physiotherapist, and dietitian, that is, when your treatment plan for the surgery began to be planned
  - What expectations did you have for the meeting?
  - What information did you receive?
  - How involved were you in the planning leading up to the surgery?
  - Did you have the opportunity to ask questions?
  - Do you feel that you understood what the treatment would lead to?
- How do you experience the collaboration around you?
- Did you have the opportunity to influence the planning?
  - In what way have you felt involved?
  - Were you able to participate in choosing different treatment options?
  - Has there been anything that was difficult to decide on?
    - How did you handle it?
- What has happened since the first meeting? [intervention day]
  - How has the time leading up to the surgery been?
  - What did/does the treatment mean to you?
  - What have you done while waiting? [for the surgery]
  - How have you experienced the period/waiting?
- Have you set any goals along the way?

**3. Closing questions**

- How are you today?
- What are you going to do after the surgery?
